# Cutaneous Metastasis in Colorectal Cancer: A Rare and Overlooked Phenomenon

**DOI:** 10.7759/cureus.53437

**Published:** 2024-02-02

**Authors:** Chinedum Okafor

**Affiliations:** 1 Pathology, Louisiana State University Health System, Shreveport, USA

**Keywords:** immunostaining, aggressive metastasis, rare presentation, cutaneous metastasis, metastatic colon adenocarcinoma, skin lesions, skin cancers, adenocarcinoma of colon, skin nodule, metastatic colon cancer

## Abstract

Cutaneous metastasis from internal malignancies, indicative of poor prognosis, is rare and often involves primary sources like lung, breast, and colorectal cancer (CRC). This case details a 66-year-old male developing a scalp lesion 10 years post colon adenocarcinoma diagnosis. The challenging medical journey included a comprehensive biopsy confirming metastatic CRC in cutaneous tissue through CDX2 and CK20 positivity, emphasizing the importance of advanced diagnostic techniques. Despite medical advancements, the patient's unfavorable prognosis led to succumbing within a year, highlighting challenges in managing such cases and the need for vigilant post-diagnosis care. This report underscores the limited understanding of cutaneous metastasis, emphasizing the role of immunostaining and prompting awareness for early detection and tailored treatment. Further research into atypical metastasis mechanisms is crucial for improved prognostic outcomes and enhanced comprehension of these complex manifestations.

## Introduction

The occurrence of metastatic cancer to the skin constitutes approximately 5.3% of all cases [1,2]. Predominantly originating from malignancies of the breast and lung [1,3-7], skin metastases are often characterized by well-differentiated, mucin-secreting adenocarcinomas [5,7]. While this phenomenon is relatively infrequent, its implications are profound, particularly in cases of metastatic colon adenocarcinoma, where the presentation to the skin can be both complex and atypical.

Clinically, metastatic colon adenocarcinoma may manifest as nodules, papules, or plaques on the skin [2,4,6,7]. The diverse presentation poses a challenge in differentiation, often mimicking various benign conditions. This mimicry, coupled with the rarity of cutaneous metastasis from colon adenocarcinoma, can contribute to misdiagnosis or delayed diagnosis, subsequently leading to delayed treatment and, consequently, poorer outcomes.

Research suggests that most metastatic adenocarcinomas to the skin follow a well-differentiated pattern [5]. This unique histologic characteristic, combined with the complex clinical presentation, underscores the intricacies involved in diagnosing metastatic colon adenocarcinoma in the cutaneous tissue. The challenges are further compounded by the potential for these tumor deposits to be misconstrued as benign conditions, emphasizing the critical need for heightened clinical suspicion and comprehensive diagnostic approaches.

Moreover, the prognosis associated with metastatic colon adenocarcinoma to the skin is notably variable, with reported survival rates ranging from one to 34 months following the diagnosis of the metastatic tumor [1]. This wide range underscores the heterogeneity of outcomes in such cases, emphasizing the significance of early and accurate diagnosis in influencing patient survival. There is, thus, a need for clinicians to educate and empower patients on skin examination, detection of any changes, and early reporting of any skin changes, especially in individuals with a history of visceral malignancy.

In light of these complexities, this study aims to contribute to the understanding of metastatic colon adenocarcinoma to the skin, shedding light on diagnostic challenges, histological features, and the impact on patient outcomes. By delving into these intricacies, this study strives to enhance clinical awareness, facilitate timely intervention, and ultimately improve prognostic outcomes for individuals affected by this rare manifestation of metastatic cancer.

## Case presentation

This case involves a 66-year-old white male who initially sought medical attention due to bleeding in his rectum. Subsequent positron emission tomography-computed tomography (PET-CT) imaging revealed significant uptake in the mid-ascending colon, prompting a right hemicolectomy. The pathology review unveiled a low-grade, well-differentiated adenocarcinoma with mucinous features originating from the cecum. The tumor exhibited invasion into the pericolic adipose tissue and adhesion to the transverse colon, classified as stage T4bN0M0.

A remarkable nine years later, the patient underwent CT imaging for a suspected biliary tract infection, uncovering a 6.7 x 4.8 cm hypodensity in segment seven of the liver. A biopsy confirmed metastatic adenocarcinoma with mucinous features, leading to the initiation of chemotherapy.

Ten years post the initial colon cancer diagnosis, the patient presented to the outpatient clinic with a right parietal scalp lesion characterized by rapid growth and easy bleeding, particularly upon irritation. Upon examination, the lesion exhibited a 2.5 cm area with overlying crusting and surrounding tender telangiectasias. The oral cavity and oropharynx displayed no lesions or masses.

**Figure 1:**
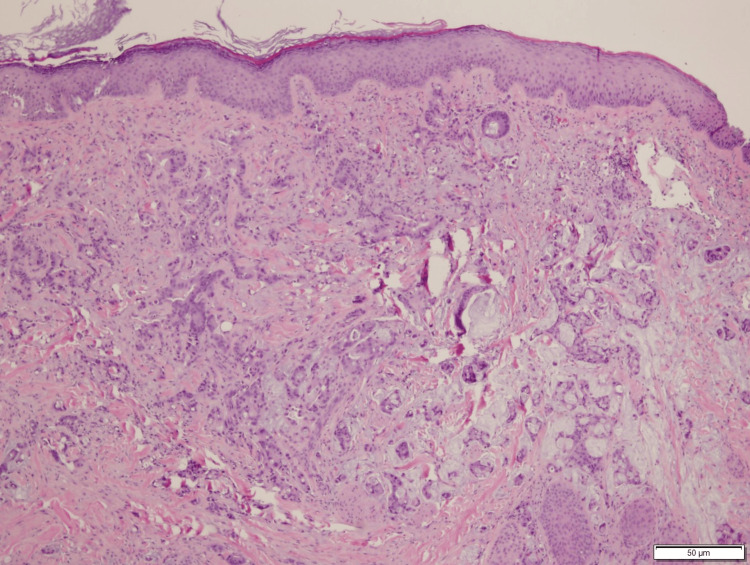
Skin with metastatic adenocarcinoma involving the dermis and adnexa Histopathological examination shows tumor cells forming irregular tubules with bluish mucin, background mucin, and overlying epidermis without an in-situ component.

**Figure 2:**
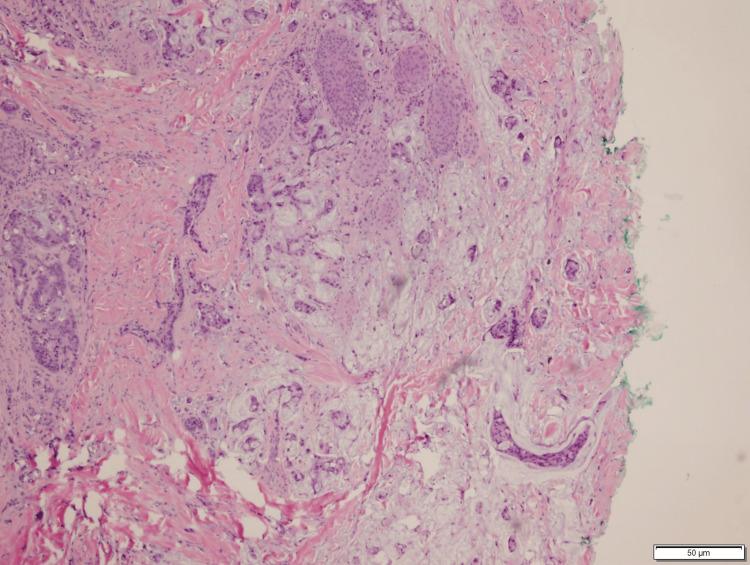
Skin with metastatic adenocarcinoma extending to the margin Tumor cells show complex and irregular tubules affecting the dermis with desmoplasia and background mucin.

A biopsy of the scalp lesion revealed a histopathological composition dominated by complex and irregular tubules with an advancing edge, encircled by desmoplasia, and exhibiting luminal and extravasated bluish mucin. Infiltration extended into the dermis, adnexa, and deep margins (see Figures 1, 2). The final diagnosis confirmed metastatic adenocarcinoma with changes consistent with a primary origin in the colon, extending to the margins.

**Figure 3:**
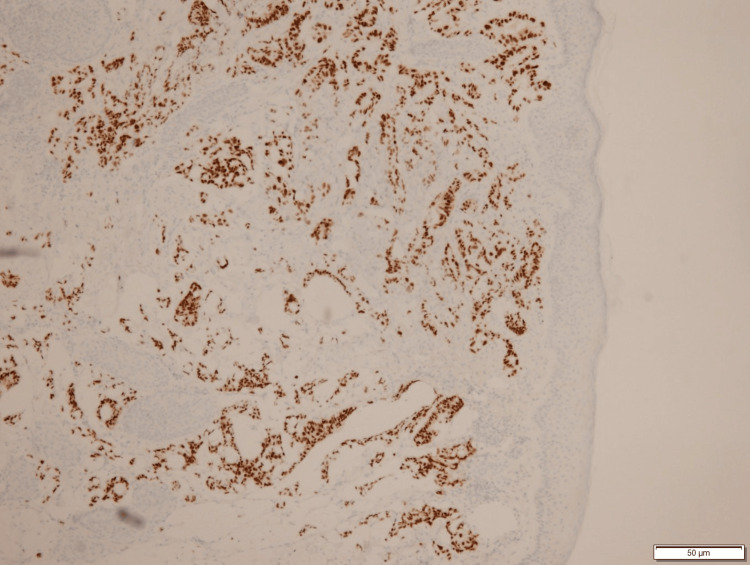
Tumor cells are positive for CDX2 consistent with colon primary There is positive staining of the tumor cells, and the overlying epidermis is negative for CDX2. This is consistent with a colon primary.

**Figure 4:**
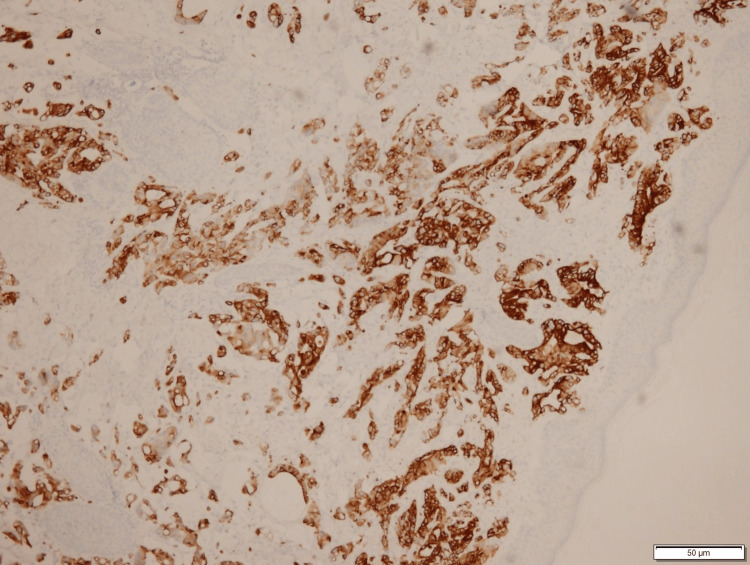
Tumor cells are positive for CK20 Tumor cells are positive for CK20, which is consistent with colon primary.

Immunohistochemistry played a pivotal role in affirming the diagnosis, with tumor cells exhibiting positivity for CDX-2 (Figure 3) and CK-20 (Figure 4), consistent with a colon primary. This distinctive presentation highlights the complex and atypical nature of cutaneous metastasis from colon adenocarcinoma, emphasizing the importance of thorough histopathological evaluation and immunostaining for accurate diagnosis.

The challenging journey of this patient underscores the need for heightened clinical awareness, especially in cases where metastases manifest in unexpected locations. Further research into the underlying mechanisms and prognostic implications of such atypical metastases is crucial for advancing our understanding and improving patient outcomes.

## Discussion

Metastasis of cancer to the skin is a rare finding, with an overall incidence of 5.3% ​[2]. Most skin metastases occur in the vicinity of the primary tumor. Cancers of the breast and lung are the most common to metastasize to the skin, usually the skin of the chest wall. Cancers of the gastrointestinal and genitourinary systems metastasize to the abdomen ​[2,3]. Scalp metastases are more common in patients with renal cell carcinoma. Cutaneous metastases, wherein cancer cells disseminate from internal organs to the skin, are not a primary presentation of malignancies. Instead, they often emerge as a secondary development, signaling an aggressive underlying cancer. Therefore, clinicians need to incorporate this possibility into their differential diagnosis when assessing skin lesions, particularly given its significance as a prognostic factor for advanced malignancy. Colorectal adenocarcinoma has an incidence of approximately 50 per 100,00 cases. Metastatic colon adenocarcinoma to the skin is an infrequent yet fascinating deadly manifestation of cancer spread. They carry a better prognosis compared to other skin metastasis from other cancers and metastasize to the perineal region and abdominal wall (periumbilical area) ​[4]. These lesions typically originate in the colon's epithelial lining and commonly metastasize to organs like the liver, lungs, and bones. However, when it spreads to the skin, it unveils a unique and perplexing aspect of its aggressiveness.

Skin presentation varies from the most commonly painless flesh-colored skin nodules with or without surrounding skin changes​ [1,3,5,6]. They can be multiple or single, like in our case, which appeared as a single parietal scalp lesion and can favor sites of previous incisions such as a colostomy site. Metastasis to the skin of the scalp can occur.

The underlying mechanism of this process is poorly understood, and most researchers suggest that the procedure may involve the cancer cells gaining access to the bloodstream and lodging themselves within cutaneous blood vessels or lymphatic channels. Another proposed mechanism points toward direct invasion of the surrounding tissues by the primary tumor, leading to the formation of secondary tumors in the skin ​[7,8]. The patient's condition deteriorated rapidly, leading to his demise. Histological results confirmed the presence of colon adenocarcinoma and the cutaneous nodules identified as dermo hypodermic metastases originating from the colon adenocarcinoma​ [8].​

This discussion highlights the rarity of cutaneous metastases, which typically indicate widespread disease and poor prognosis, with varying survival rates. Although colorectal cancer (CRC) is common, cutaneous metastases from this cancer type are exceedingly unusual and often manifest within two years of primary tumor resection. The locations of cutaneous involvement align with the prior tumor site, but distant metastases can occur. The appearance of cutaneous metastases can mimic various skin conditions, necessitating histological evaluation for accurate diagnosis. Immunostaining with markers like CK20 and CDX2 is essential for differentiation as CRC typically expresses CK20 and CDX2.

## Conclusions

In conclusion, a meticulous examination and assessment of skin lesions in patients with a confirmed history of colon cancer, coupled with a high index of suspicion, are imperative. This scrutiny not only influences therapeutic decisions but also holds significant implications for patient outcomes. The occurrence of metastasis from colon cancers to the face is an exceedingly rare phenomenon, often eluding detection for extended periods and associated with a dismal prognosis. Therefore, maintaining a vigilant awareness of this possibility should be a priority, particularly in individuals with a known diagnosis of colon cancer. Accurate diagnosis demands a thorough histological evaluation, and the inclusion of immunostaining with markers such as CK20 and CDX2 proves indispensable for precise differentiation.

In essence, this study underscores the rarity of facial metastasis from colon cancer, emphasizing the need for heightened clinical suspicion, thorough diagnostic approaches, and proactive patient education. Healthcare providers play a pivotal role in empowering individuals to recognize any skin changes, and encouraging them to report these changes promptly is crucial for early detection and intervention. By fostering a comprehensive understanding of this infrequent yet clinically significant phenomenon, clinicians can contribute to enhanced detection, timely intervention, and improved overall care for individuals navigating the complexities of metastatic colon cancer.
